# Prescriptions for insulin and insulin analogues in children with and without major congenital anomalies: a data linkage cohort study across six European regions

**DOI:** 10.1007/s00431-023-04885-6

**Published:** 2023-03-04

**Authors:** Joanne Given, Joan K. Morris, Ester Garne, Elisa Ballardini, Laia Barrachina-Bonet, Clara Cavero-Carbonell, Mika Gissler, Francesca Gorini, Anna Heino, Sue Jordan, Amanda J. Neville, Anna Pierini, Ieuan Scanlon, Joachim Tan, Stine K. Urhoj, Maria Loane

**Affiliations:** 1 Faculty of Life & Health Sciences, Ulster University, Belfast, Northern Ireland UK; 2 Population Health Research Institute, St George’s, University of London, London, UK; 3 Pediatric Department, Hospital Lillebaelt, Kolding, Denmark; 4 Neonatal Intensive Care Unit, Paediatric Section, Dep. of Medical Sciences, IMER Registry, University of Ferrara, Ferrara, Italy; 5 Rare Diseases Research Unit, Foundation for the Promotion of Health and Biomedical Research in the Valencian Region, Valencia, Spain; 6 THL Finnish Institute for Health and Welfare, Helsinki, Finland; 7 Unit of Epidemiology of Rare Diseases and Congenital Anomalies, Institute of Clinical Physiology, National Research Council, Pisa, Italy; 8 Faculty of Medicine, Health and Life Science, Swansea University, Swansea, Wales UK; 9 IMER Registry (Emilia Romagna Registry of Birth Defects), Center for Clinical and Epidemiological Research, University of Ferrara, Ferrara, Italy; 10 Paediatric Department, Hospital Lillebaelt, Kolding, Denmark and Section of Epidemiology, Department of Public Health, University of Copenhagen, Copenhagen, Denmark

**Keywords:** Cohort study, Congenital anomalies, Data linkage, Down syndrome, Diabetes Mellitus requiring insulin

## Abstract

Are children with major congenital anomalies more likely to develop diabetes requiring insulin therapy, as indicated by prescriptions for insulin, than children without congenital anomalies? The aim of this study is to evaluate prescription rates of insulin/insulin analogues in children aged 0–9 years with and without major congenital anomalies. A EUROlinkCAT data linkage cohort study, involving six population-based congenital anomaly registries in five countries. Data on children with major congenital anomalies (60,662) and children without congenital anomalies (1,722,912), the reference group, were linked to prescription records. Birth cohort and gestational age were examined. The mean follow-up for all children was 6.2 years. In children with congenital anomalies aged 0–3 years, 0.04 per 100 child-years (95% CIs 0.01–0.07) had > 1 prescription for insulin/insulin analogues compared with 0.03 (95% CIs 0.01–0.06) in reference children, increasing ten-fold by age 8–9 years. The risk of > 1 prescription for insulin/insulin analogues aged 0–9 years in children with non-chromosomal anomalies (RR 0.92, 95% CI 0.84–1.00) was similar to that of reference children. However, children with chromosomal anomalies (RR 2.37, 95% CI 1.91–2.96), and specifically children with Down syndrome (RR 3.44, 95% CIs 2.70–4.37), Down syndrome with congenital heart defects (RR 3.86, 95% CIs 2.88–5.16) and Down syndrome without congenital heart defects (RR 2.78, 95% CIs 1.82–4.27), had a significantly increased risk of > 1 prescription for insulin/insulin analogues aged 0–9 years compared to reference children. Female children had a reduced risk of > 1 prescription aged 0–9 years compared with male children (RR 0.76, 95% CI 0.64–0.90 for children with congenital anomalies and RR 0.90, 95% CI 0.87–0.93 for reference children). Children without congenital anomalies born preterm (< 37 weeks) were more likely to have > 1 insulin/insulin analogue prescription compared to term births (RR 1.28, 95% CIs 1.20–1.36).

*Conclusion:* This is the first population-based study using a standardised methodology across multiple countries. Males, children without congenital anomalies born preterm and those with chromosomal anomalies had an increased risk of being prescribed insulin/insulin analogues. These results will help clinicians to identify which congenital anomalies are associated with an increased risk of developing diabetes requiring insulin therapy and allow them to reassure families of children who have non-chromosomal anomalies that their risk is similar to that of the general population.

**Table:** 

**What is Known:** *• Children and young adults with Down syndrome have an increased risk of diabetes requiring insulin therapy.* *• Children born prematurely have an increased risk of developing diabetes requiring insulin therapy.*	
**What is New:** *• Children with non-chromosomal anomalies do not have an increased risk of developing diabetes requiring insulin therapy compared to children without congenital anomalies.* *• Female children, with or without major congenital anomalies, are less likely to develop diabetes requiring insulin therapy before the age of 10 compared to male children.*

**What is Known:**

*• Children and young adults with Down syndrome have an increased risk of diabetes requiring insulin therapy.*

*• Children born prematurely have an increased risk of developing diabetes requiring insulin therapy.*

**What is New:**

*• Children with non-chromosomal anomalies do not have an increased risk of developing diabetes requiring insulin therapy compared to children without congenital anomalies.*

*• Female children, with or without major congenital anomalies, are less likely to develop diabetes requiring insulin therapy before the age of 10 compared to male children.*

## Introduction

Congenital anomalies (CAs) (structural defects and chromosomal abnormalities) are a leading cause of infant mortality, morbidity, and long-term disability. Little is known about the risk of co-morbidities in children with CAs. The EUROlinkCAT project aims to investigate prescription rates of medications for chronic diseases as a measure of co-morbidity in children with CAs [1]. This study focuses on insulin/insulin analogue prescriptions used to treat diabetes in childhood. The most common type of diabetes requiring insulin therapy in children is type 1 diabetes mellitus, with monogenic forms of diabetes affecting just 1.1–4.2% [2, 3] of those with childhood diabetes.

Historically, a number of case reports and small scale cross-sectional studies reported a higher prevalence of type 1 diabetes among those with Down syndrome than in the general population [4–8]. However, these studies had a number of methodological issues including small highly selected samples, reliance on questionnaires with low response-rates and urinalysis to diagnose diabetes. More recently, a population-based study using registry data in Denmark (1981–2000) found a four-fold increased risk of type 1 diabetes in those with Down syndrome aged between 2 and 22 years compared with the non-Down syndrome group (Odds Ratio (OR) 4.12, 95% CI 2.1– 8.2) [9]. A subsequent German study using diabetes registries reported that the onset of type 1 diabetes occurred during the first 3 years of life in 18.9% of Down syndrome patients with type 1 diabetes and in 6.4% of those with type 1 diabetes without Down syndrome [10]. Other genetic anomalies, such as Klinefelter syndrome [11] and Turner syndrome [12–14] have also been linked with type 1 diabetes.

A case–control study in Sweden found that patients with type 1 diabetes and congenital heart defects (CHD) had an earlier onset of diabetes compared with patients with type 1 diabetes without CHD (mean 13.9 versus 17.4 years, p < 0.001) [15]. A subsequent cohort study by the same group found that patients with CHD born 1970–1984 had an increased risk of type 1 diabetes (HR 1.87, 95% CI 1.56–2.24), but not for those born 1985–1993 (HR 1.14, 95% CI 0.91–1.42), compared with matched controls [16].

Monogenic diabetes, which includes neonatal diabetes, maturity-onset diabetes of the young (MODY) and rare forms of syndromic diabetes, are caused by one or more defects in a single gene [17, 18]. Genetics are estimated to contribute to 50% of the risk of developing type 1 diabetes [19] but numerous environmental influences have also been implicated.

The risk of diabetes requiring insulin therapy among children with CAs has not previously been examined in a large sample, in multiple regions/countries using a standardised methodology. In this paper, we examine prescriptions of insulin and insulin analogues, as an indicator of diabetes requiring insulin therapy, in six European regions over a 15-year period for children with CAs compared with a cohort of reference children without CAs [1].

## Methods

EUROlinkCAT is a European, population-based linkage cohort study including data from six European Surveillance of Congenital Anomalies (EUROCAT) registries (https://eu-rd-platform.jrc.ec.europa.eu/eurocat_en), in five countries. Live born children with a major CA recorded in each EUROCAT registry born between 2000 and 2014 were included, although not all registries covered the complete time period: Denmark: Funen (2000–2014), Finland (2000–2014), Italy: Emilia Romagna (2008–2014), Italy: Tuscany (2008–2014), Spain: Valencian Region (2010–2014) and UK: Wales (2000–2014). Live born children without CAs born during the same time-period and from the same population area covered by the registry were included as a reference group. Reference children covering the whole population were available for all registries, apart from Tuscany, which had a sample of 10% of the reference population, matched on year of birth and sex. All children born at 23 weeks or more gestational age were included in the study (in Wales reference children born at 24 weeks or more were included).

### Classification of CAs

CAs were classified according to the EUROCAT anomaly subgroups [20] using the International Classification of Diseases, Ninth or Tenth Revision—British Paediatric Association codes [ICD9-BPA or ICD10-BPA]. CAs are coded using codes beginning with 74–75 in ICD-9, and codes in the Q-chapter of ICD-10. Children with only minor anomalies, defined as anomalies with lesser medical, functional or cosmetic consequences, according to the EUROCAT definitions were excluded [20]. Children with metabolic or endocrine disorders are not included in EUROCAT. Isolated anomalies are defined as anomalies within a single organ, as defined using the EUROCAT algorithm [20]. Isolated CAs with sufficient insulin/insulin analogue exposed child-years to be included in analysis were CHD [ICD10-BPA] Q20-Q26), cleft lip with or without cleft palate (Q36,Q37), cleft palate (Q35), congenital hydronephrosis (Q62.0), club foot/talipes equinovarus (Q66.0), hip dislocation and/or dysplasia (Q65.0-Q65.2, Q65.80, Q85.81), and craniosynostosis (Q75.0). Non-isolated CAs with sufficient insulin/insulin analogue exposed child-years to be included in analysis were chromosomal anomalies (Q90-Q92, Q93, Q96-Q99), Down syndrome (Q90), Down syndrome with CHD (Q90 with Q20-Q26) and Down syndrome without CHD (Q90 without Q20-Q26).

### Classification of insulin exposure

Prescriptions issued (UK, Wales) or dispensed (all other registries) were recorded in the prescription databases using the WHO Anatomical Therapeutic Chemical (ATC) classification. Insulin/insulin analogues are recorded using ATC codes starting with A10A. A child must have had at least two prescriptions in a single year to be classified as exposed to insulin or insulin analogues. Restricting the analysis to at least two prescriptions for insulin/insulin analogues reduces the risk of data entry errors inflating the proportion of children who are considered to have diabetes.

### Electronic prescription databases and linkage

Information on prescriptions issued or dispensed up to a child’s 10^th^ birthday (or 31^st^ December 2015) was available by linking to local prescription databases, see Supplemental Table S1. Data on prescriptions were included from 1^st^ January 2000 (or the first birth year with linked medication data available for each registry) until 31^st^ December 2015. This allowed at least one year of follow-up information for each child. Two registries followed-up children from birth to 7 years (Emilia Romagna, and Tuscany) and one followed-up children from birth to 5 years (Valencian Region). The remaining three registries had information on at least some children from birth to 9 years of age.

### Data standardisation

EUROCAT data on CAs were already standardized [20]. Prescription data in each participating registry were standardized to a common data model and a central analysis script produced aggregate tables for analysis [1]. The aggregate tables were uploaded to a secure portal for download by the study team for pooled analysis. Individual data on children remained at local registry level. Reference children were identified from birth records. Both reference children and children with CAs could only be linked to a prescription record if they had a valid ID number.

### Small numbers

Four registries (Denmark: Funen, UK: Wales, Italy: Tuscany and Italy: Emilia Romagna) have rules for releasing data with small numbers from their linked databases. The Secure Anonymised Information Linkage (SAIL) databank (UK: Wales) provided data to the EUROlinkCAT Central Results Repository based at Ulster University with the requirement that any individual counts involving one to four children would not be published. Denmark: Funen and Italy: Emilia Romagna provided data with the requirement that any individual results involving fewer than five children would not be released and Italy: Tuscany provided data with the requirement that any individual results involving fewer than three children would not be released.

### Statistical methods

The number of children in the population, number of child-years of follow-up, number with at least two insulin/insulin analogue prescriptions/dispensations per year and prevalence per 100 child-years was calculated for each year of age (for example birth to 1^st^ birthday, one year of age to 2^nd^ birthday etc.). To avoid potential disclosure issues, ages were grouped where necessary into 0 to 3 years, 4 to 5 years, 6 to 7 years, 8 to 9 years and 0 to 9 years.

Random effects meta-analysis was used to pool the prevalence of insulin/insulin analogue prescriptions using the Freeman-Tukey Double Arcsine Transformation to stabilize the variances of the proportions. Random effects meta-analysis was used to combine the relative risk (RR) of 2 or more prescriptions from each registry for children with CAs compared with reference children. Heterogeneity between registries was assessed by Cochran (Q) and I^2^ statistics, which expressed the percentage of variation between registries.

As rates of insulin/insulin analogue prescriptions increased with age and there were differences between registries, only those registries that had children with ten years of follow-up (Finland, Denmark: Funen and UK: Wales) were included in the analysis investigating the risk of insulin/insulin analogue prescriptions for children with specific anomalies. To comply with statistical disclosure controls, only anomalies with a total of > 5 exposed child-years were examined. The number of child-years of follow-up and number with at least two insulin/insulin analogue prescriptions/dispensations each year were summed for the three registries and used to calculate the prevalence of insulin/insulin analogue prescription per 100 child-years and the RR compared with reference children for each anomaly. The data were summed as the continuity corrections, which were necessary due to the rarity of anomalies and insulin exposures in the age groups included in this study, greatly influenced the RRs estimated from the standard meta-analytic procedures.

### Analysis of risk factors

We examined the effect of birth cohort (births in 2000–2004 compared with 2005–2009) on risk of insulin/insulin analogue prescriptions in reference children and children with CAs. The 0 to 9 age group could not be used as the 2000–2004 birth cohort was the only one to have all children followed up for 9 years in the 3 regions with births starting in 2000 (Finland, Denmark: Funen and UK: Wales). We chose the 0 to 3 years age group as all children in both birth cohorts had follow-up to at least 4 years of age. The effect of being born in 2010–2014 was not examined as those born at the end of the cohort were not followed up for the full 4 years. RRs were calculated after summing the number of child-years of follow-up and number of children with at least two insulin/insulin analogue prescriptions/dispensations each year.

We also examined the effect of gestational age (< 37 weeks compared with ≥ 37 weeks) and sex (female compared with male) on risk of insulin/insulin analogue prescriptions in reference children and children with CAs from 0 to 9 years. RRs were calculated after summing the number of child-years of follow-up and number with at least two insulin/insulin analogue prescriptions/dispensations each year, in each risk factor category, for the registries with ten years of follow-up (Finland, Denmark: Funen and UK: Wales).

All statistical analyses were performed using Stata version 16.0 (StataCorp LP, College Station, TX, USA).

## Results

**Fig. 1:**
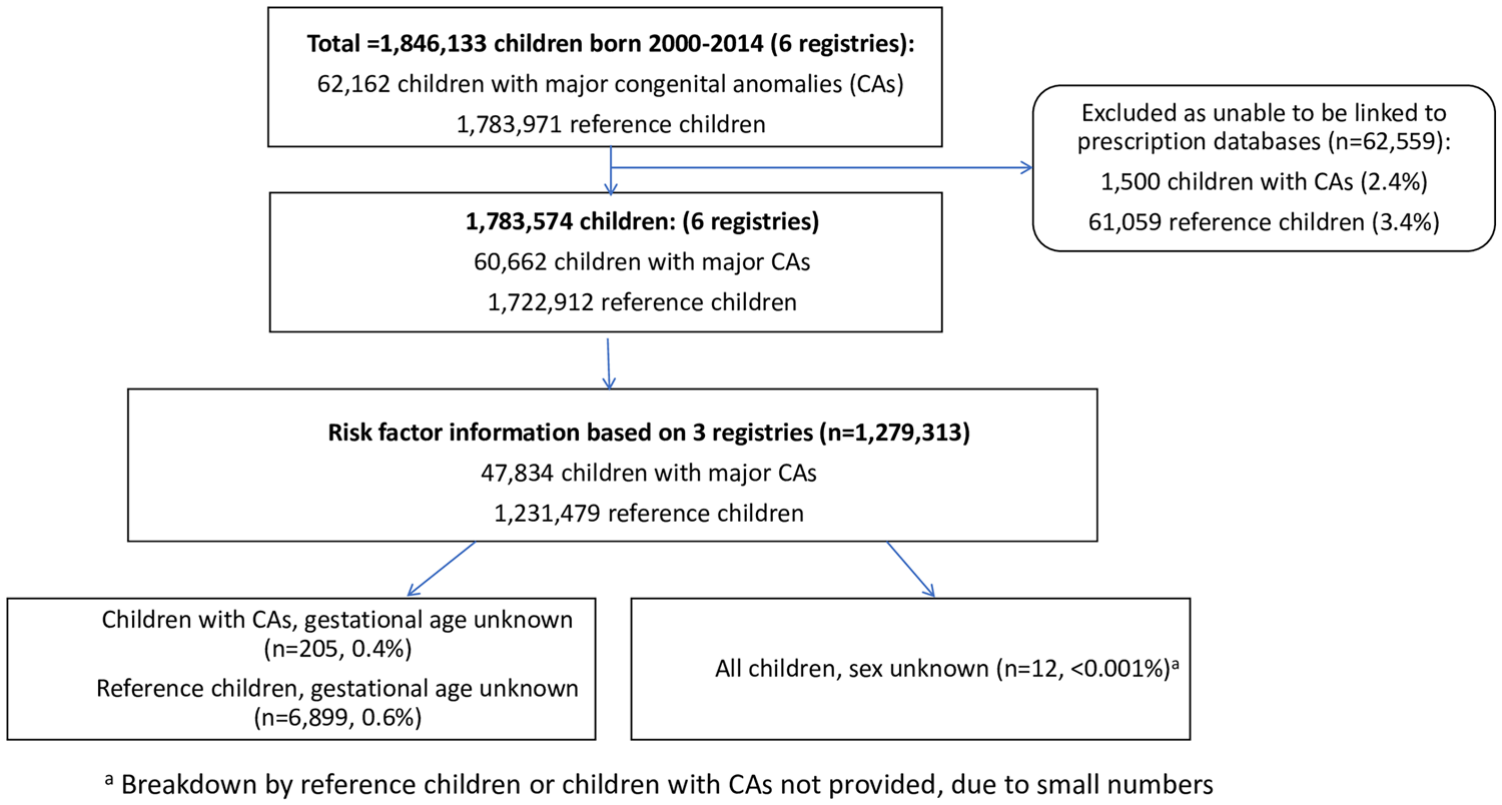
Total number of children born in the six regions, number included in the analysis and number with missing risk factor information

The study population comprised 60,662 children with major CAs and 1,722,912 reference children without CAs, (Fig. 1). Together Finland and Wales contributed 67.6% of the population. Children with CAs were followed-up for 376,166 child-years and reference children for 10,707,343 child-years. Mean follow-up for both children with CAs and reference children was 6.2 years. Three registries had data on children up to their 10^th^ birthday, of which 18,898 were children with CAs (31.2% of all children with CAs) and 532,411 were reference children (30.9% of all reference children).

**Fig. 2:**
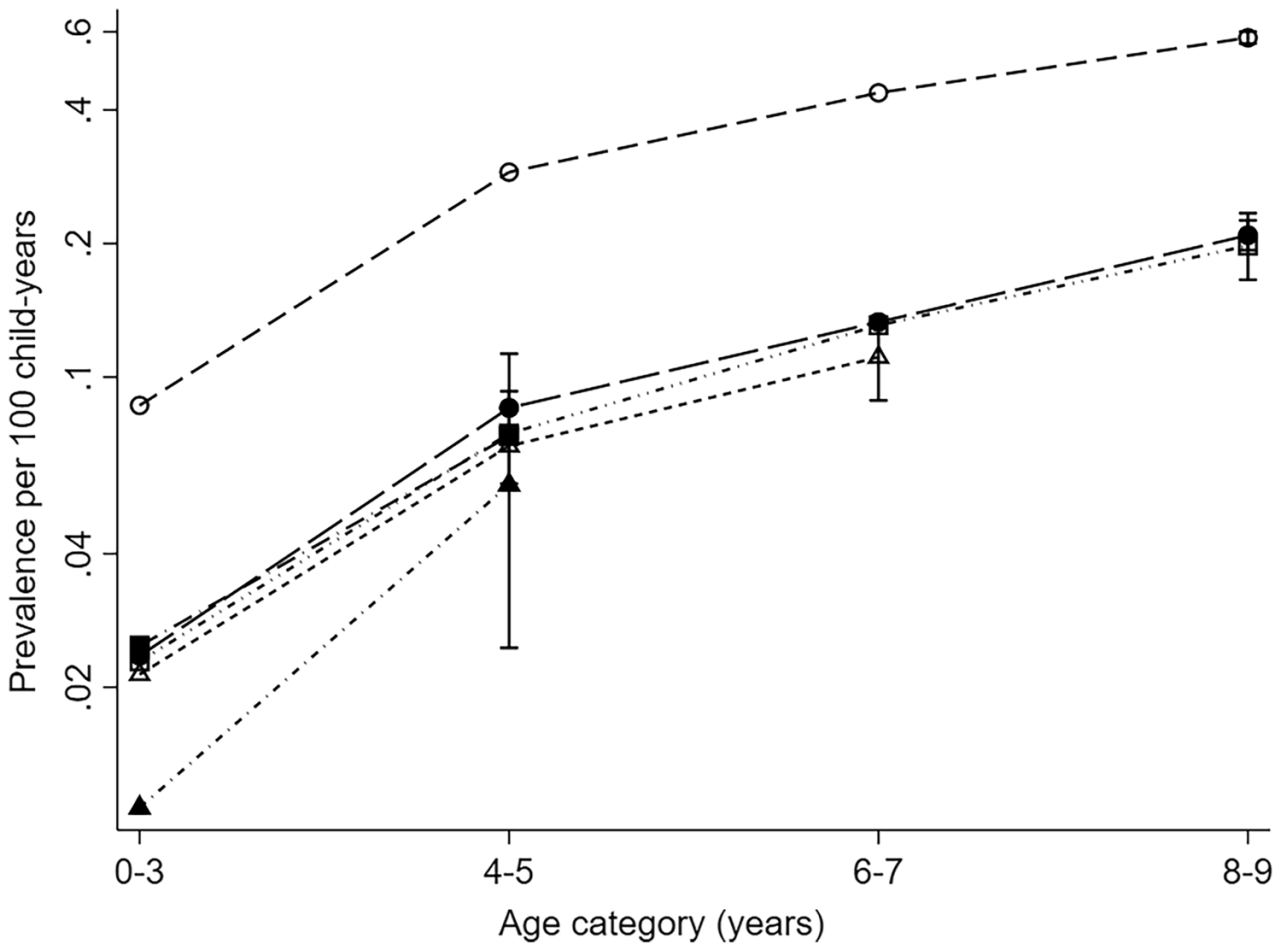
Prevalence per 100 child-years of insulin/insulin analogue prescriptions at 0 to 3, 4 to 5, 6 to 7 and 8 to 9 years of age, and 95% CIs at last follow-up period (log scale), among reference children in each registry. White circle = Finland; Black circle = UK: Wales; White square = Denmark: Funen; Black square = Spain: Valencian Region; White triangle = Italy: Emilia Romagna; Black Triangle = Italy: Tuscany

Among children with CAs and reference children, the prevalence of > 1 insulin/insulin analogue prescription increased with age in all registries. At 4 to 5 years, the oldest age group with data for all registries, the prevalence of > 1 prescription for insulin/insulin analogues was lowest for reference children in Italy: Tuscany (0.06, 95% CI 0.02–0.11 per 100 child-years) and highest in Finland (0.29, 95% CI 0.28–0.30 per 100 child-years). This pattern continued into the older age groups (Fig. 2) (prevalence in Tuscany age 6 to 7 years not shown due to small numbers). The same pattern of prescriptions was observed in children with CAs, with the prevalence being much higher for children in Finland than in other registries. Prevalence by registry is not shown for children with CAs as some registries/age groups had ≤ 5 child-years with insulin/insulin analogue exposures.

### Meta-analysis (all registries combined)

In children with CAs, there was > 1 prescription for insulin/insulin analogues in 0.04 per 100 child-years (95% CI 0.01–0.07, heterogeneity I^2^ 90.4%, p < 0.001) at 0 to 3 years of age compared with 0.03 per 100 child-years (95% CI 0.01–0.06, heterogeneity I^2^ 99.6%, p < 0.001) in the reference group. This increased to 0.40 per 100 child-years (95% CI 0.22–0.63) among those with CAs aged 8 to 9 years and 0.31 per 100 child-years (95% CI 0.10–0.63) in the reference group. Children with CAs were more likely to have > 1 prescription for insulin/insulin analogues than reference children in all of the age groups examined, but none of these increases were statistically significant (Table 1).

**Table 1:** Number of child-years with > 1 insulin/insulin analogue prescription, prevalence of insulin prescription per 100 child-years (95% CIs) aged 0 to 3, 4 to 5, 6 to 7, 8 to 9 years (2000–2014) and Risk Ratio for exposure in children with CAs compared with reference children

Age group	Reference children		Children with CAs		Children with CAs compared with reference children
	Child-years with > 1 prescription	Prevalence per 100 child-years (95% CIs)	Child-years with > 1 prescription	Prevalence per 100 child-years (95% CIs)	Risk Ratio (95% CIs)
0 to 3 years	3,168	0.03 (0.01–0.06)	130	0.04 (0.01–0.07)	1.46 (0.77–2.78)
4 to 5 years	3,947	0.10 (0.03–0.20)	143	0.12 (0.04–0.22)	1.16 (0.76–1.78)
6 to 7 years^a^	4,648	0.16 (0.05–0.33)	166	0.18 (0.06–0.37)	1.14 (0.78–1.65)
8 to 9 years^b^	4,773	0.31 (0.10–0.63)	163	0.40 (0.22–0.63)	1.24 (0.77–2.01)

*CAs *Congenital Anomalies, *CI *Confidence Interval

^a^All registries excluding Spain: Valencian Region

^b^Includes Finland, UK: Wales and Denmark: Funen

### Specific subgroups of CAs

**Fig. 3:**
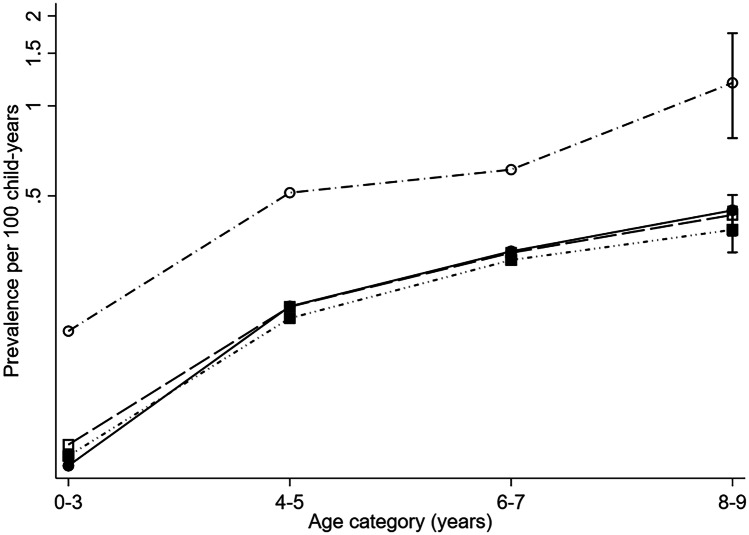
Prevalence per 100 child-years of insulin/insulin analogue prescription at 0 to 3, 4 to 5, 6 to 7 and 8 to 9 years of age, and 95% CIs at 8 to 9 years, with insulin/insulin analogue prescription (log scale), among reference children, all CAs, Chromosomal and non-chromosomal CAs in Denmark: Funen; Finland and UK: Wales. White circle = Chromosomal anomalies; Black circle = Reference children; White square = All CAs; Black square = Non-chromosomal anomalies. CAs = Congenital Anomalies

We found a significantly increased risk of receiving > 1 prescription for insulin/insulin analogues 0 to 9 years of age among children with chromosomal anomalies (RR 2.37, 95% CI 1.91–2.96), and specifically in children with Down syndrome (RR 3.44, 95% CI 2.70–4.37), Down syndrome with CHD (RR 3.86, 95% CI 2.88–5.16) and Down syndrome without CHD (RR 2.78, 95% CI 1.82–4.27) compared to reference children (Table 2 and Fig. 3). The risk of receiving > 1 prescription for insulin/insulin analogues in children 0 to 9 years of age with non-chromosomal (RR 0.92, 95% CI 0.84–1.00) anomalies is similar to that of the reference children. Only children with congenital hydronephrosis were found to have a significantly decreased risk (RR 0.57, 95% CI 0.35–0.92) of receiving > 1 prescription for insulin/insulin analogues aged 0 to 9 years.

**Table 2:** Number of children, number of child-years with > 1 insulin/insulin analogue prescription, prevalence of insulin/analogue prescription per 100 child-years (95% CIs) and Risk Ratio for > 1 insulin/insulin analogue prescription among CAs with > 5 exposed child-years compared with reference children in Denmark: Funen; Finland and UK: Wales (0–9 years)

	Number of children	Child-years with > 1 prescription	Prevalence per 100 child-years (95% CIs)	Risk Ratio compared with reference children (95% CIs)
Reference children	1,231,479	15,852	0.18 (0.18–0.19)	NA
All CAs	47,834	593	0.18 (0.17–0.20)	1.00 (0.92–1.08)
Non-chromosomal anomalies	44,964	513	0.17 (0.15–0.18)	0.92 (0.84–1.00)^a^
CHD	15,637	185	0.18 (0.15–0.20)	0.97 (0.84–1.12)
Cleft lip with or without cleft palate	992	9	0.13 (0.06–0.24)	0.69 (0.36–1.32)
Cleft palate	968	20	0.28 (0.17–0.44)	1.55 (1.00–2.40)^b^
Congenital hydronephrosis	2,410	17	0.10 (0.06–0.17)	0.57 (0.35–0.92)
Club foot	1,532	19	0.17 (0.10–0.26)	0.93 (0.59–1.46)
Hip dislocation and/or dysplasia	838	6	0.09 (0.03–0.21)	0.52 (0.23–1.15)
Craniosynostosis	527	10	0.28 (0.13–0.51)	1.53 (0.82–2.84)
Chromosomal anomalies	2,868	80	0.43 (0.34–0.54)	2.37 (1.91–2.96)
Down syndrome	1,507	66	0.63 (0.48–0.80)	3.44 (2.70–4.37)
Down syndrome with CHD	909	45	0.70 (0.51–0.94)	3.86 (2.88–5.16)
Down syndrome without CHD	598	21	0.51 (0.31–0.78)	2.78 (1.82–4.27)

*CAs *Congenital Anomalies, *CI *Confidence Interval, *CHD *Congenital Heart Defect

^a^0.999 before rounding

^b^> 1.002 before rounding

### Risk factors for diabetes

Children born in 2000–2004 had a similar risk of receiving > 1 prescription for insulin/insulin analogues aged 0 to 3 as those born in 2005–2009; the RR was 1.04 (95% CI 0.66–1.62) for children with CAs, and 1.03 (95% CI 0.94–1.12) for reference children.

Children with CAs born at < 37 weeks gestational age had a 24% decreased risk of being issued/dispensed > 1 prescription aged 0 to 9 years (RR 0.76, 95% CI 0.58–0.99) compared with children born at ≥ 37 weeks which was borderline statistically significant. In reference children the opposite effect was seen as the risk of being issued/dispensed > 1 prescription was increased 28% (RR 1.28, 95% CI 1.20–1.36) in children born at < 37 weeks compared with those born ≥ 37 weeks gestation.

Female children had a reduced risk of being issued/dispensed > 1 prescription aged 0 to 9 years compared with male children (RR 0.76, 95% CI 0.64–0.90 for children with CAs and RR 0.90, 95% CI 0.87–0.93 for reference children).

### Sensitivity analysis

When the criterion of at least two prescriptions for insulin/insulin analogues to indicate type 1 diabetes was relaxed to at least one prescription for insulin/insulin analogues, the prevalence among children with CAs increased slightly from 0.08 to 0.09 (95% CI 0.03–0.17) per 100 child-years by the end of follow-up (mean 6.2 years). There was no change from 0.07 per 100 child-years by the end of follow-up (mean 6.2 years) for reference children.

## Discussion

This is the first population-based study to examine insulin/insulin analogue prescriptions in children with all major CAs, and specific CAs, compared with reference children. As expected, we found increasing prevalence with increasing age. There was evidence for considerable heterogeneity among regions in terms of the prevalence of insulin/insulin analogue prescriptions for both reference children and children with CAs. This is expected as the incidence rate for type 1 diabetes, which will account for most cases of childhood diabetes requiring insulin therapy, varies markedly between countries [21]. In Europe there is a north–south gradient in the incidence of type 1 diabetes [22], with Finland having the highest incidence of type 1 diabetes in childhood in Europe [23, 24] which is consistent with our findings.

The prevalence of insulin/insulin analogue prescriptions among all children with CAs was not statistically significantly different to that seen among reference children. However, children with chromosomal anomalies, specifically children with Down syndrome, were at an increased risk of > 1 insulin/insulin analogue prescription compared with reference children. This finding is in agreement with previous studies based on crude measures of diabetes [4, 5, 9] or small sample sizes [4, 5, 7]. An earlier population-based study identified 8 children with Down syndrome and type 1 diabetes out of 2,094 children with Down syndrome which corresponded to a 4.2 fold increased prevalence compared with the background population [9]. Our findings of a 3.4 fold increased prevalence corroborate this. Beta cell autoantibodies have been identified in Down syndrome patients with type 1 diabetes supporting an autoimmune cause of diabetes in at least a proportion of children with Down syndrome and type 1 diabetes [10, 25]. Parents of children with chromosomal anomalies should be made aware of the increased risk of developing diabetes and should be informed of the symptoms of diabetes so that they are aware of these.

Children with CHD were not at an increased risk of > 1 insulin/insulin analogue prescription compared with reference children aged 0 to 9 years. The two previous studies in Sweden which explored type 1 diabetes among those with CHD did not use standardized CA registry data. Instead, they used the National Patient Register on hospitalizations (inpatient and outpatient diagnoses) or death certificates and included a range of non-CHD diagnoses in the ICD codes used to identify CHD cases, such as secondary hypertension (which may be secondary to diabetes) and vitium organicum cordis [16]. The CHD population will therefore have included some non-CHD cases and those with minor anomalies, such as patent ductus arteriosus in pre-term infants and foramen ovale, which are excluded from EUROCAT data. The increased risk of developing type 1 diabetes among those with CHD born in 1970–1984, but not among those born 1985–1993 [16], may also reflect better recording of both CHD and type 1 diabetes in more recent years.

This study highlights the difficulty of exploring a rare disease among children with rare anomalies. It is only through pooling data from several countries or regions, such as performed in this EUROlinkCAT study, that we were able to examine the risk of diabetes requiring insulin therapy for a number of anomalies not previously described in the literature. It was not possible to examine the risk of receiving > 1 prescription for insulin/insulin analogues in children with Klinefelter and Turner syndrome due to the rarity of these anomalies and the small number that were born alive. Future studies should include additional countries and years of follow-up to allow an examination of risk in rare congenital anomalies. If data on screening and genetic testing were available, it may also be possible to distinguish between type 1 diabetes and monogenic diabetes in children less than one year old in future studies. In our study, all children had at least 1 year of follow-up, yet the prevalence of being issued/dispensed > 1 prescription for insulin/insulin analogues was lowest in children 0–3 years. It is possible that children with chromosomal anomalies may have an increased risk of requiring insulin therapy given the genetic origins of monogenic diabetes, but given the rarity of monogenic diabetes, affecting 1–4% of childhood diabetes, it is unlikely that this will have affected our results on children with chromosomal anomalies. The decreased risk of receiving > 1 prescription for insulin/insulin analogues among those with congenital hydronephrosis has not previously been reported and may be a chance finding due to the number of comparisons made. It should be confirmed in other data sources before children with congenital hydronephrosis are considered to truly have a decreased risk of requiring insulin therapy.

The prevalence of > 1 insulin/insulin analogue prescription in reference children and in children with CAs aged 0 to 3 years born between 2000–2004 was not statistically significantly different to the prevalence rates for children born 2005–2009. Based on a large multicentre European study 1989–2013, Patterson et al. reported a 3.7% per annum increase in incidence rate of type 1 diabetes in both boys and girls aged 0–4 years. In the same study, they also reported a possible slowing down of increasing incidence among children under 15 years of age in the 2004–2008 period. In particular, the increase in incidence rates in high-incidence countries such as Finland and two out of three UK centres (Oxford and Northern Ireland) started to abate [26]. Harjutsalo et al. found that the previously increasing incidence (1988–2005) of type 1 diabetes in children under 15 years of age in Finland had plateaued in the most recent years (2005–2011) [27]. The fact that it was only possible to examine the change in prevalence over time in the 0–3 year age group in the earlier years may also have contributed to the failure to find any evidence for increasing prevalence rates over time, as the incidence of type 1 diabetes peaks in puberty [28].

As per the literature, reference children born < 37 weeks gestational age have a higher risk of > 1 insulin/insulin analogue prescription than those born at term. Preterm birth has previously been associated with increased risk of developing type 1 diabetes [29]. The higher risk of type 1 diabetes in preterm born children may be explained by reduced insulin sensitivity [30], gut dysbiosis [31], exposure to antenatal corticosteroids [32] and rapid weight gain in infancy [33] due to catch up growth [34]. Some forms of neonatal diabetes are associated with in utero insulin secretory insufficiency and growth retardation [35] which may in turn lead to elective preterm delivery [36]. Our study included children born from 23 weeks gestational age, so those born very preterm were included. Preterm children with CAs had a reduced risk of > 1 insulin/insulin analogue prescription compared with children with CAs born at term, which was of borderline significance. This may reflect the small sample size or slower weight gain in infancy in these children due to the impact of their anomalies [37, 38].

Type 1 diabetes does not show a strong female bias, unlike many other common autoimmune diseases such as hyperthyroidism, thyroiditis, rheumatoid arthritis, and multiple sclerosis [39]. The incidence of type 1 diabetes peaks in puberty, which occurs in girls earlier than boys, but the follow-up period was just short of this [28]. In adults, males and females have the same prevalence of type 1 diabetes and it may be the case that the reduced risk for females seen here would not be present were the sample followed up to early adulthood. However, the prevalence is slightly higher in adult males in the USA, Denmark and Sweden and adult females in Japan, Australia and Africa [22, 39].

The main strength of this study is the population-based setting. Information is available on over 1.78 million children with valid ID numbers that allowed children to be linked to their prescriptions, from six European regions, in five countries covering both Northern and Southern Europe. In addition, the EUROCAT registries have a high level of case ascertainment and use standardized definitions and coding of CAs to ensure consistency across Europe. The use of reference children for comparison enables interpretation of the results for children with CAs in the context of results for unaffected children. In five of the six regions, reference children represented 100% of the national/regional population. Finally, this study used electronic prescription records for insulin/insulin analogues as a proxy for diabetes rather than depending on diagnoses recorded in electronic hospital/medical records. It is widely accepted that the quality of electronic prescription records is good, especially if these have been established for a number of years, as is the case in our study (e.g., electronic prescriptions in earlier years for Valencian Region, Spain, were not included in this study, as there were known data quality issues).

A potential limitation of this study is that we do not have access to hospital prescribing, as some children may have been prescribed insulin/insulin analogues at hospital. However, if a child has been diagnosed with diabetes requiring insulin therapy, then that child will use insulin for the rest of his/her life, and these prescriptions are issued in primary care. Therefore, we are confident that we are not overestimating diabetes requiring insulin therapy, though we may miss some in younger age groups if these children got their prescriptions in hospital. Finland and Wales accounted for two-thirds of the data, so data from these countries heavily influence the results and may not be representative of Europe as Finland has the highest prevalence of type 1 diabetes in Europe, and Wales has one of the highest rates of child poverty in Western Europe. Also, we did not have complete follow-up to the child’s 10^th^ birthday for all children in the study.

This is the first population-based study to use a standardised methodology to examine prescribing of insulin/insulin analogues in children with all CAs, and a range of specific CAs, compared with reference children. While all children with CAs were not at increased risk of diabetes requiring insulin therapy, children with specific chromosomal anomalies, particularly children with Down syndrome and CHD, had an increased risk. The results will help clinicians to identify which congenital anomalies are associated with an increased risk of developing diabetes requiring insulin therapy and allow them to reassure families of children who have non-chromosomal anomalies that their risk is similar to that of the general population.

## Data Availability

The study data are available from the authors for scientifically valid requests and with the permission of the participating registries. https://www.eurolinkcat.eu/contactinformationanddatarequests.

## Abbreviations

ATC: Anatomical Therapeutic Chemical
CA: Congenital anomalies
CHD: Congenital heart defects
EUROCAT: European Surveillance of Congenital Anomalies
MODY: Maturity-onset diabetes of the young
ICD10-BPA: International Classification of Diseases, Tenth Revision - British Paediatric Association
UK: United Kingdom
